# Prevalence of Vitamin D Deficiency in Patients With Charcot Arthropathy: A Single-Center Analysis

**DOI:** 10.5435/JAAOSGlobal-D-22-00162

**Published:** 2022-10-21

**Authors:** Robert Daniel Kay, Johan Forslund, D.'Ann Arthur, Adam James Taylor, Arash Aminian

**Affiliations:** From Department of Orthopaedic Surgery Residency Office, Harbor-University of California, Los Angeles, Medical Center, Los Angeles, CA.

## Abstract

Vitamin D deficiency may be a potentially modifiable risk factor in patients with orthopaedic conditions including Charcot arthropathy. The purpose of this study was to determine the prevalence of vitamin D deficiency and insufficiency in patients with Charcot arthropathy.

**Methods:**

All patients with Charcot arthropathy seen in our foot and ankle surgery clinic from January 2017 through June 2021 were screened for serum 25-hydroxyvitamin D levels. Patients were categorized as sufficient, insufficient, or deficient based on previously accepted guidelines. The prevalence of vitamin D deficiency and insufficiency was calculated.

**Results:**

A total of 57 subjects were included in this study after meeting the inclusion criteria. Of these, 27 (47.4%) were found to be deficient in vitamin D, 21 (36.8%) were insufficient in vitamin D, and 9 (15.8%) were sufficient in vitamin D. Overall, 84.2% of the cohort was found to be either insufficient or deficient in vitamin D.

**Conclusion:**

Vitamin D insufficiency and deficiency is highly prevalent in patients with Charcot arthropathy. As such, it is possible that this may play a role in the pathogenesis of Charcot arthropathy and may represent a potentially modifiable risk factor that could be optimized during the management of patients with Charcot arthropathy.

Vitamin D is recognized as an essential component in bone heath. The 2011 Centers for Disease Control and Prevention report on vitamin D levels in Americans found that 8% of the cohort was deficient and another 25% were at risk for insufficient levels based on recommended daily values.^1^

Vitamin D deficiency is estimated to affect more than 1 billion people globally.^2^ In recent years, several studies have shown a high prevalence of vitamin D deficiency in some cohorts of orthopaedic patients,^3,4^ including patients with certain foot and ankle disorders.^5,6,7,8^ To our knowledge, the prevalence of vitamin D deficiency has not been reported on or well established in patients with Charcot arthropathy. The purpose of this study was to identify the prevalence of vitamin D deficiency in patients with Charcot arthropathy.

Charcot arthropathy occurs in patients with neuropathy and may lead to significant bony destruction and deformity of the foot and ankle. It is well recognized that one of the most common underlying etiologies of Charcot arthropathy is diabetes mellitus, which leads to significant damage of the peripheral nerves.^9^ The precise incidence of Charcot neuroarthropathy in persons with diabetes has been previously estimated to be between 0.1% and 0.4%.^10,11^ One etiology of the Charcot process is increased bone turnover due to autonomic dysfunction and increased blood flow through arteriovenous shunting.^9^ Bone turnover markers have been found to be elevated in patients with acute Charcot neuroarthropathy compared with controls.^12^ In patients with Charcot arthropathy, bone density analysis confirms the presence of osteopenia and indicates an increased risk for neuropathic fractures.^13,14^

Vitamin D is a key modulator of bone remodeling, making it an important factor during periods of high bone turnover such as the acute Charcot process or following fractures. Through its action with osteoblasts, vitamin D enhances osteoclastic function and plays a major role in bone remodeling and mineralization.^15^ Its effect has been reported in the literature as increasing bone mineral density and preventing fragility fractures in the elderly.^16^

Patients with Charcot may be challenging to treat given their significant deformity, bone loss, increased body habitus, and medical comorbidities. Given the mechanism of action of vitamin D, vitamin D deficiency may be a metabolic factor in the progression of deformity, fractures, and postoperative failure of fixation in patients with Charcot arthropathy. Therefore, optimization of this modifiable risk factor could improve the possibility of positive outcomes for patients with Charcot in a cost-efficient manner.

To our knowledge, there are no studies that specifically evaluate vitamin D status in patients with Charcot arthropathy. We hypothesize that there is a high prevalence of vitamin D deficiency and insufficiency in patients with Charcot arthropathy. Should this be the case, this is a potentially modifiable nutritional risk factor for this patient cohort.

## Methods

We performed an institutional review board–approved retrospective review of patients seen in our safety net hospital orthopaedic foot and ankle surgery clinic with Charcot arthropathy from January 2017 through June 2021. Inclusion criteria included all patients with active or chronic Charcot arthropathy. Exclusion criteria included all patients with previously known diagnoses of vitamin D deficiency or insufficiency and patients who had recently been on vitamin D supplementation. During the study period, vitamin D levels (serum 25-hydroxyvitamin D) were drawn on all consecutive patients with both acute and chronic Charcot arthropathy.

For the purposes of this study, active Charcot arthropathy was defined as any patient with acute swelling and warmth of the foot and ankle with radiographic foot and ankle fractures at initial presentation. At the time of serum vitamin D collection, these patients were being treated in a total contact cast. Chronic Charcot arthropathy was defined as any patient with no swelling or warmth of the foot and ankle, radiographs with mature fractures, and stable deformity. At the time of vitamin D collection, these patients had advanced to treatment in a Charcot Restraint Orthotic Walker boot, controlled ankle motion boot, or shoe. Demographic data including age, sex, body mass index, ethnicity, and medical comorbidities were also collected.

Vitamin D deficiency and insufficiency were defined as serum 25-hydroxyvitamin D levels <20 and 20 to 29 ng/mL, respectively, whereas vitamin D sufficiency was defined as serum 25-hydroxyvitamin D levels ≥30 ng/mL (Table 1). This was based on the Endocrine Society's latest guidelines.^17^ Patients who were found to be vitamin D deficient or insufficient were started on a supplemental dosage of 1,000 international units (IUs) of vitamin D_3_ based on work by Holick^15^ and referred to their primary care physicians for further monitoring and management. The vitamin D dosage was based on the recommended dietary intake of vitamin D in adults from the National Osteoporosis Foundation and Institute of Medicine.^18^ The prevalence of vitamin D deficiency and insufficiency was then tabulated.

**Table 1:** Vitamin D Level Classification System

Vitamin D Status	Serum 25-Hydroxyvitamin D Level (ng/mL)
Vitamin D deficient	<20
Vitamin D insufficient	20-29
Vitamin D sufficient	≥30

Vitamin D classification based on recommendations from the Endocrine Society.^17^

## Results

A total of 57 subjects were identified who met the inclusion criteria and were included in this study. Of the 57 patients, 31 (54.4%) were male, and 26 (45.6%) were female. The mean age of the patients was 53.3 ± 7.92 (range 39 to 78) years. The mean body mass index of the patients was 33.28 ± 7.24 (range 21.6 to 50.62) kg/m^2^. At the time of vitamin D collection, 16 patients (28.1%) had active Charcot process and 41 (71.9%) had chronic Charcot arthropathy. A list of specific medical comorbidities of each patient can be seen in Table 2. All 57 patients (100%) had an underlying diagnosis of diabetes mellitus.

**Table 2:** Patient Demographics, Medical Comorbidities, Stage of Charcot Disease Process, and Vitamin D Levels

Patient	Gender	Age	BMI	Medical Comorbidities	Active Versus Chronic Charcot	Serum 25-Hydroxyvitamin D (ng/mL)	Vitamin D Level Classification
1	F	53	40.6	DM	Chronic	21.8	Insufficient
2	M	57	38.4	DM, HTN, cirrhosis, and hepatocellular carcinoma	Chronic	22.7	Insufficient
3	M	54	23.9	DM and HTN	Chronic	34.4	Sufficient
4	F	46	26.1	DM, HTN, and HLD	Chronic	13.1	Deficient
5	F	51	26.7	DM	Chronic	20.4	Insufficient
6	F	47	21.6	DM, HTN, and ESRD on HD	Chronic	27.2	Insufficient
7	M	50	34.6	DM, HTN, and CHF	Chronic	16.4	Deficient
8	F	42	29.5	DM	Chronic	18.4	Deficient
9	M	54	24.2	DM and ESRD on HD	Active	23.2	Insufficient
10	M	42	27.1	DM	Active	28.8	Insufficient
11	F	52	40.4	DM, HTN, HLD, CKD, CHF, and GERD	Chronic	13.0	Deficient
12	F	64	36.0	DM	Chronic	12.0	Deficient
13	F	59	32.7	DM, HTN, and HLD	Active	14.0	Deficient
14	M	43	23.1	DM, HTN, and ESRD on HD	Chronic	35.0	Sufficient
15	M	45	50.6	DM, HTN, HLD, OSA, and venous stasis	Chronic	8.0	Deficient
16	M	78	26.8	DM, HTN, and HLD	Active	13.0	Deficient
17	M	63	27.8	DM and CAD	Chronic	10.0	Deficient
18	F	58	35.6	DM, HTN, and HLD	Chronic	29.0	Insufficient
19	M	63	31.1	DM and CKD	Chronic	12.0	Deficient
20	M	44	30.8	DM	Chronic	20.0	Insufficient
21	F	59	38.6	DM and HLD	Chronic	15.0	Deficient
22	F	47	35.8	DM, HTN, and HLD	Chronic	11.0	Deficient
23	M	50	48.3	DM, HTN, HLD, and CHF	Active	14.0	Deficient
24	M	45	25.6	DM and HTN	Active	39.0	Sufficient
25	M	47	29.9	DM	Active	32.0	Sufficient
26	M	50	32.7	DM, HTN, HLD, and CKD	Active	30.0	Sufficient
27	M	48	33.7	DM, HTN, and HLD	Active	24.0	Insufficient
28	F	45	48.0	DM, HTN, HLD, CHF, ESRD on HD, and papillary thyroid cancer s/p thyroidectomy	Active	11.0	Deficient
ī29	M	56	28.3	DM and HTN	Active	12.0	Deficient
30	M	68	27.5	DM, HTN, HLD, CAD, and COPD	Chronic	22.0	Insufficient
31	M	50	49.2	DM, HTN, HLD, CKD, and CHF	Chronic	26.0	Insufficient
32	M	52	38.2	DM and HTN	Chronic	30.0	Sufficient
33	F	61	37.1	DM, HTN, and HLD	Chronic	18.0	Deficient
34	F	66	27.5	DM, HLD, cirrhosis, and hepatocellular carcinoma	Chronic	21.0	Insufficient
35	F	39	30.8	DM	Active	20.0	Insufficient
36	F	57	33.9	DM, HTN, CHF, atrial fibrillation, and hypothyroidism	Chronic	19.0	Deficient
37	F	43	31.9	DM	Chronic	18.4	Deficient
38	F	51	31.2	DM and HLD	Active	17.0	Deficient
39	F	61	34.5	DM, HTN, HLD, and CKD	Chronic	22.0	Insufficient
40	F	52	44.5	DM and HTN	Chronic	14.0	Deficient
41	F	65	45.5	DM, HTN, and ESRD on HD	Chronic	13.0	Deficient
42	M	43	28.2	DM	Chronic	23.0	Insufficient
43	M	46	27.1	DM	Chronic	27.0	Insufficient
44	F	53	31.3	DM	Chronic	13.0	Deficient
45	F	63	25.6	DM, HTN, CAD, CHF, ESRD on HD, and atrioventricular block with pacemaker	Chronic	33.0	Sufficient
46	M	49	34.9	DM and HTN	Chronic	11	Deficient
47	M	45	30.0	DM and HTN	Active	32	Sufficient
48	M	53	30.7	DM	Active	16	Deficient
49	M	58	35.2	DM and ESRD on HD	Active	38	Sufficient
50	M	61	47.3	DM, HLD, HTN, BPH, and OSA	Active	17	Deficient
51	M	65	26.4	DM and HLD	Active	14	Deficient
52	F	52	32.3	DM	Chronic	26	Insufficient
53	M	55	22.9	DM, HTN, HLD, and PAD	Active	24	Insufficient
54	F	55	34.6	DM	Active	28	Insufficient
55	M	50	42.1	DM, HTN, and HLD	Active	28	Insufficient
56	F	60	39.1	DM, HTN, HLD, and GERD	Active	17	Deficient
57	M	53	29.3	DM, HTN, HLD, and CKD	Chronic	21	Insufficient

BMI = body mass index, CAD = coronary artery disease, CHF = congestive heart failure, CKD = chronic kidney disease, COPD = chronic obstructive pulmonary disease, DM = diabetes mellitus, ESRD = end-stage renal disease, GERD = gastroesophageal reflux disease, HD = hemodialysis, HLD = hyperlipidemia, HTN = hypertension, OSA = obstructive sleep apnea, PAD = peripheral arterial disease

Patients ordered in a chronological order by which they presented to our clinic.

**Figure 1:**
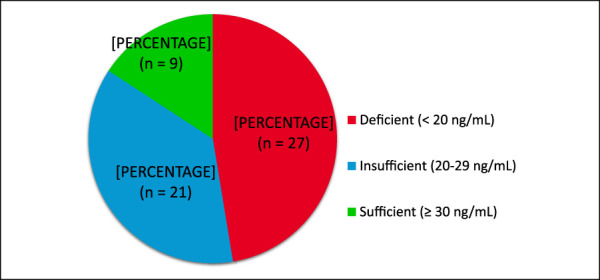
Pie chart showing the prevalence of vitamin D deficiency, insufficiency, and sufficiency in patients with Charcot arthropathy.

The mean vitamin D level among all patients was 20.86 ± 7.8 ng/mL (range 8–39 ng/mL), with 27 patients (47.4%) found to be vitamin D deficient, 21 patients (36.8%) vitamin D insufficient, and 9 patients (15.8%) vitamin D sufficient (Figure 1). Overall, 84.2% of the patients were either insufficient or deficient in vitamin D. Specific vitamin D levels for each patient are listed in Table 2.

## Discussion

Vitamin D is involved in the maintenance of healthy bone metabolism through regulation of calcium and phosphate homeostasis.^19^ Untreated vitamin D deficiency can result in bone diseases such as osteoporosis, rickets, and osteomalacia. Recent studies have shown a high prevalence of vitamin D deficiency in orthopaedic patients.^19,20^ Smith et al evaluated vitamin D levels in low-energy foot and ankle fracture patients compared with patients with ankle sprains and no fractures. They found that 60% of fracture patients were insufficient or deficient in vitamin D, and vitamin D levels were statistically significantly higher in patients who had ankle sprains with no fractures.^8^ They suggest the possible link between vitamin D levels and propensity for fracture. In a prospective cohort study, Aujla et al^5^ found that 82.5% of 577 patients undergoing elective foot and ankle surgery were deficient or insufficient in vitamin D. Horas et al^7^ reported that 84% of 31 patients with bone marrow edema syndrome of the foot and ankle were either deficient or insufficient in vitamin D. Fraissler et al^6^ evaluated the prevalence of vitamin D deficiency in 65 patients with osteochondral injuries of the talus and found that 75% of the patients had either deficient or insufficient levels of vitamin D. In a retrospective review of 126 revision total hip and knee arthroplasty patients, Traven et al^21^ identified vitamin D deficiency as a modifiable risk factor for reducing complications after revision arthroplasty. In addition, studies have shown vitamin D deficiency to be associated with notably worse outcomes in patients undergoing total knee arthroplasty.^22,23^

In our current study, we report on patients with Charcot arthropathy who were referred to our orthopaedic foot and ankle surgery clinic from January 2017 through June 2021. Consistent with the prior orthopaedic literature on fracture care, arthroplasty, and foot and ankle diseases, we found a high rate of vitamin D insufficiency and deficiency among our patients with Charcot foot deformity and fractures. Specifically, 84.2% of the patient cohort was either insufficient or deficient in vitamin D. As such, our study identifies a potentially modifiable independent risk factor in patients with Charcot arthropathy that may affect the management of these patients and the ability to treat the disease.

Although our patients were referred to their primary care provider for definitive management of hypovitaminosis D, a brief review of the literature regarding management may be helpful for orthopaedic providers aiming to treat these deficiencies. The Endocrine Society Clinical Practice Guideline^17^ suggests that adequate vitamin D intake for the average adult is 600 to 800 IU/d, although this may not be enough to maintain serum 25-hydroxyvitamin D above 30 ng/mL. We opted to preliminarily start our patients with hypovitaminosis D on 1,000 IU/d as suggested by Holick^15^ and refer them to their primary care provider for definitive management. An example of management for vitamin D deficiency presented by the Endocrine Society involves 50,000 IU of vitamin D_2_ or D_3_ once a week for 8 weeks or 6,000 IU daily until serum 25-hydroxyvitamin D is above 30 ng/mL, followed by maintenance therapy of 1,500 to 2,000 IU daily.^17^ Management strategies vary based on patient factors, and detailed treatment algorithms for vitamin D deficiency are beyond the scope of this study.

Vitamin D in the body is derived from dietary sources and through synthesis in the skin after exposure to ultraviolet radiation.^24^ The biologically active form of vitamin D is 1,25(OH)2-vitamin D_3_ (calcitriol). This is derived from the biologically inactive form of vitamin D_3_ (cholecalciferol), which is hydroxylated to 25(OH)-vitamin D_3_ (calcifediol) in the liver and again hydroxylated to the active form 1,25(OH)2-vitamin D_3_ (calcitriol) in the kidneys.^24^ Once converted to calcitriol, the active form of vitamin D, its effect on bone metabolism is through the regulation of serum calcium and phosphate. In addition, vitamin D receptors on chondrocytes and osteoblasts have been found to stimulate endochondral ossification, leading to calcification of the osteoid matrix produced by osteoblasts.^25^ Thus, deficiency in vitamin D may result in diseases related to mineral incorporation such as rickets and osteomalacia.

Given the involvement of the kidneys in the processing of vitamin D, patients with impaired renal function often have impaired vitamin D metabolism known as renal osteodystrophy. This can result in low serum 25-hydroxyvitamin D and calcitriol levels, in addition to vitamin D resistance due to loss of vitamin D receptors in the parathyroid gland and impaired binding of vitamin D to the vitamin D receptors.^26^ In the current study, 13 patients (23%) had documented renal dysfunction (chronic kidney disease or end-stage renal disease on hemodialysis). Of these patients, 4 (31%) were deficient, 5 (38%) were insufficient, and 4 (31%) were sufficient in vitamin D. Although not all the renal-impaired patients were deficient in vitamin D, it is important to recognize that even with sufficient serum vitamin D levels these patients may still be functionally deficient in vitamin D because of vitamin D resistance at the level of the vitamin D receptors and also the inability to create the active form 1,25(OH)2-vitamin D_3_ (calcitriol) in the kidneys. Patients with renal impairment who are deficient in vitamin D are often treated with the active form 1,25(OH)2-vitamin D_3_ (calcitriol) or a related analog for these reasons.^27^ These patients may suffer from primary or secondary osteoporosis in addition to primary or secondary hyperparathyroidism, all factors that may contribute to the previously established increased prevalence of Charcot arthropathy in renal-impaired patients.^28^

Our study has several limitations. The data presented involve a single-center analysis representing patients from a single geographic region. The geographic location and the amount of ultraviolet light exposure may affect vitamin D levels, potentially making our results less generalizable to differing geographic locations. Furthermore, we did not analyze the dietary or supplementation habits (aside from vitamin D supplementation) in our cohort, and as such, there was likely a wide range of dietary vitamin D intake. However, the risk for selection bias within the confines of a single-center study was minimized by including all comers with Charcot arthropathy not previously treated with vitamin D supplementation. In addition, as many risk factors contribute to the Charcot process, a larger sample size and a multivariate analysis of multiple variables or a matched control group would be necessary to further prove that vitamin D deficiency is a risk factor or associated with Charcot arthropathy. This was beyond the scope of the current observational study in which the focus was on identifying the prevalence of vitamin D deficiency or insufficiency in the Charcot arthropathy cohort. Another limitation is that we did not evaluate how vitamin D supplementation affects the management of Charcot arthropathy. Further studies are needed to assess the efficacy of vitamin D therapy regarding outcomes in patients with Charcot arthropathy. Finally, our study was based out of a safety net county hospital, and as such, the patients who presented may have been a more marginalized cohort that was already at risk of vitamin D deficiency due to nutritional deficits.

## Conclusion

Vitamin D deficiency and insufficiency were highly prevalent among our patients with Charcot arthropathy. In this study, 84.2% of patients with Charcot arthropathy presenting to the orthopaedic foot and ankle surgery clinic over a 4-year period were found to be either insufficient or deficient in vitamin D. As such, it is possible that vitamin D may play a role in the pathogenesis of Charcot arthropathy. Given that vitamin D is essential for generalized bone health and for the bone's ability to recover from insult, vitamin D represents a potentially modifiable risk factor that could be optimized during both surgical and nonsurgical management of patients with Charcot arthropathy.
